# Genetic architecture of common bunt resistance in winter wheat using genome-wide association study

**DOI:** 10.1186/s12870-018-1435-x

**Published:** 2018-11-13

**Authors:** Amira M. I. Mourad, Ahmed Sallam, Vikas Belamkar, Ezzat Mahdy, Bahy Bakheit, Atif Abo El-Wafaa, P. Stephen Baenziger

**Affiliations:** 1 Department of Agronomy and Horticulture, University of Nebraska–Lincoln, Lincoln, NE USA; 2 Department of Agronomy, Faculty of Agriculture, Assiut University, Asyut, Egypt; 3 Department of Genetics, Faculty of Agriculture, Assiut University, Asyut, Egypt

**Keywords:** *Triticum aestivum*, Linkage disequilibrium, Marker-assisted selection, Correlation, Gene annotation

## Abstract

**Background:**

Common bunt (caused by *Tilletia caries* and *T. foetida*) has been considered as a major disease in wheat (*Triticum aestivum*) following rust (*Puccinia* spp.) in the Near East and is economically important in the Great Plains, USA. Despite the fact that it can be easily controlled using seed treatment with fungicides, fungicides often cannot or may not be used in organic and low-input fields. Planting common bunt resistant genotypes is an alternative.

**Results:**

To identify resistance genes for Nebraska common bunt race, the global set of differential lines were inoculated. Nine differential lines carrying nine different genes had 0% infected heads and seemed to be resistant to Nebraska race. To understand the genetic basis of the resistance in Nebraska winter wheat, a set of 330 genotypes were inoculated and evaluated under field conditions in two locations. Out of the 330 genotypes, 62 genotypes had different degrees of resistance. Moreover, plant height, chlorophyll content and days to heading were scored in both locations. Using genome-wide association study, 123 SNPs located on fourteen chromosomes were identified to be associated with the resistance. Different degrees of linkage disequilibrium was found among the significant SNPs and they explained 1.00 to 9.00% of the phenotypic variance, indicating the presence of many minor QTLs controlling the resistance.

**Conclusion:**

Based on the chromosomal location of some of the known genes, some SNPs may be associated with *Bt1*, *Bt6*, *Bt11* and *Bt12* resistance loci. The remaining significant SNPs may be novel alleles that were not reported previously. Common bunt resistance seems to be an independent trait as no correlation was found between a number of infected heads and chlorophyll content, days to heading or plant height.

**Electronic supplementary material:**

The online version of this article (10.1186/s12870-018-1435-x) contains supplementary material, which is available to authorized users.

## Background

Common bunt (CB) caused by *Tilletia caries* (D.C.) Tul. (=*T. tritici*) and *T. foetida* (Wallr.) Liro (=*T. laevis*) can cause huge losses in wheat grain yield. Infected plants with common bunt usually produce low grain yield with low quality compared with healthy plants. The reduction in the yield and its quality in the infected plants occurs due to the replacement of grains with bunt balls spores [1, 2]. Furthermore, wheat millers usually reject kernels infected by this pathogen as very low infection rates can result in noticeable undesirable odors in flour. In the USA-Great Plains, an area from central Texas through central Nebraska, common bunt rarely causes large yield losses. However, it has been considered as an important factor which reduces grain quality in this region [3]. Seed treatments with fungicides could be used as an effective tool to manage common bunt. However, genetic resistance is a better option for reducing exposure to chemical seed treatments and could be applied in organic systems [4, 5].

Estimation of common bunt resistance is difficult as the disease is scored at very late stages of plant development when bunt balls form during the grain filling stage (Feekes 11.3 and 11.4). Moreover, occasionally the formation of bunt balls can occur only in the last spike formed on the plant and only in a few of the florets [1]. To overcome these limitations in the direct assessment of common bunt resistance, marker-assisted selection (MAS) could be used. One of the most effective marker systems which have been used widely in plant breeding for different traits is Genotyping-by-sequencing (GBS). Genotyping-by-sequencing usually generates a lot of SNP markers that cover large genomic regions in a cost-effective manner [6, 7]. Those genome-wide SNPs could be utilized in different genomics studies including genome-wide association study (GWAS), genomic selection, and genetic diversity studies. Association mapping (AM) is a robust tool to identify alleles of interest that control the phenotypic variation among genotypes [8]. To detect alleles associated with target traits using GWAS, 100–500 individuals and codominant markers (SSR or SNP) are highly recommended for the analysis [9].

In order to apply MAS in breeding for common bunt resistance, information on the genes reducing common bunt infection is needed. Resistance to common bunt was often recorded as a quantitative trait controlled by a single gene which has a complete or incomplete dominance effect [10, 11]. Sixteen race-specific resistance genes for common bunt have been identified, from *Bt1* to *Bt15* and *Btp* [12, 13]. Some of these sixteen resistance genes have been mapped [14]. Unfortunately, no information has been published on the common bunt race in Nebraska, hence there is no information on the resistance genes against Nebraska common bunt race.

The objectives of this study are: 1) identify genes that are resistant to the Nebraska race of common bunt using 14 differential lines, 2) screen a set of 330 Nebraska winter wheat lines for resistance to common bunt in multiple locations, 3) identify alleles/genomic regions associated with common bunt resistance using GWAS, and 4) study the correlation of common bunt resistance and agronomic traits (such as chlorophyll content, days to heading, and plant height) which could possibly be used as a selection criterion.

## Results

### Evaluating the differential lines and susceptible checks

**Fig. 1:**
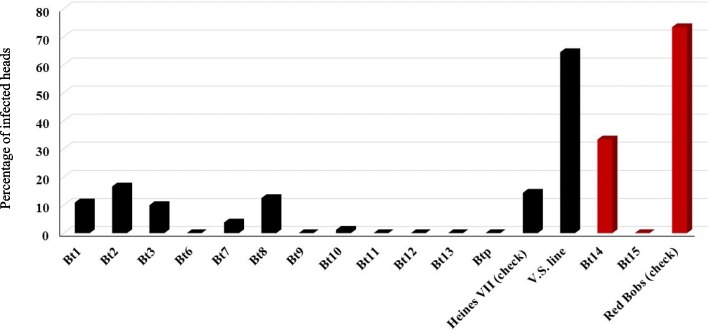
Percentage of infected heads in the common bunt differential lines set based on the average of Mead, Lincoln, and greenhouse. Black columns represent the percentage of infected heads in the winter differential lines as well as a check at the field. Red charts represent the percentage of infected heads in the spring differential lines as well as the check in the greenhouse

The common bunt differential lines, as well as the susceptible checks, were screened for their resistance to the Nebraska common bunt race in the field and greenhouse. The winter check “Heines VII” had very a low percentage of infected heads with an average infected head of 14.4% which could be interpreted as our inoculation was unsuccessful. However, number of genotypes were susceptible to highly susceptible (see below), so we believe our test is valid. While, the spring check (Red Bobs), evaluated in the greenhouse, had a high percentage of infected heads (73.5%) (Fig. 1). Out of the twelve-winter wheat differential lines, seven lines (*Bt6*, *Bt9*, *Bt11*, *Bt12*, *Bt13*, *Bt15,* and *Btp*) were very resistant to Nebraska common bunt race with zero% infected heads. In addition, two differential lines, *Bt10* and *Bt7*, were resistant and had 1.2% and 3.8%, infected heads. The remaining five lines containing *Bt1*, *Bt2*, *Bt3*, *Bt8*, and *Bt14*) had a percentage of infected heads ranging from 10 to 33.3% hence were considered moderately susceptible to susceptible.

To test the ANOVA of the common bunt resistant data, the percentage data were transformed using arcsine root square method. Compared with untransformed data (means of infection percentages), the transformed data were normally distributed (Additional file 1: Figure S1). Shapiro-Wilk normality test had a non-significant value (*p*-value 0.1024) for the transformed data, while, it was highly significant for the original data (*p*-value = 1.688e^− 05^) indicating the non-normal distribution for the original common bunt scores.

**Fig. 2:**
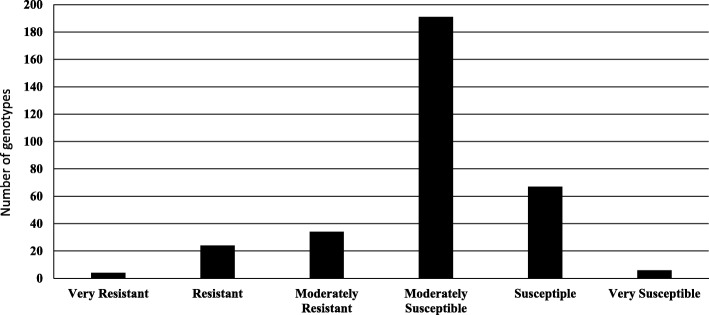
Number of genotypes showing different degrees of common bunt resistance based on the average of both locations (Mead and Lincoln)

The ANOVA for common bunt resistance revealed highly significant differences among the entries, no significant differences between the locations, and no significant Location x Entry (LxE) interaction (Table 1). Highly significant correlation between the two locations was found (*r* = 0.61, *P* < 0.01) (Additional file 2: Figure S2). The 330 tested genotypes had different percentages of infected heads ranging from 0 to 64.5% as an average of both locations. Based on these results, all genotypes could be classified into six groups namely; very resistant (four genotypes- 0%), resistant (24 genotypes – 0.1-5.0%), moderately resistant (34 genotypes – 5.1-10.00%), moderately susceptible (191 genotypes- 10.01-30.00%), susceptible (67 genotypes- 30.01-50.00%) and very susceptible (six genotypes – 50.01-100%) (Fig. 2). Broad-sense heritability was high based on the average from both locations (H^2^_B_ = 0.78).

**Table 1:** Analysis of variance, broad sense heritability and coefficient of variation (C.V.) of common bunt resistance, plant height, heading date, and chlorophyll content

Source of variance	Common bunt resistance		Plant Height		Heading date		Chlorophyll content	
	d.f.	Mean Squares	d.f	Mean Squares	d.f	Mean Squares	d.f	Mean Squares
Location	1	0.08	1	471.83*	1	41,923.56**	–	–
Replicate (Loc.)	2	0.00019	2	644.91**	2	16.87	1	18.05
Iblock (Rep.)	8	0.15	8	3290.16**	8	428.14**	8	208.50**
Pcol (iblock)	93	0.05	93	168.01**	93	41.67**	93	19.87
Prow	6	0.004	6	166.46	6	30.38*	6	13.01
Entry	328	0.49**	331	134.76**	331	28.77**	354	15.26
Location*Entry	328	0.08	331	62.64	331	11.22	–	–
Broad-sense Heritability	0.78		0.40		0.51		0.06	
C.V. (%)	48.00		12.00		1.72		9.77	

*p < 0.05, **p < 0.01

Highly significant differences were found among the tested genotypes for the remaining traits except for chlorophyll content. Broad-sense heritability of plant height and days to heading was 0.40 and 0.51, respectively. Both traits, plant height and days to heading, were normally distributed (Additional file 3: Figure S3). No correlation was found between the percentage of infected heads and plant height. However, a small negative significant correlation (*r* = − 0.11, *P* < 0.05) was found between the infected heads and days to heading (Table 2).

**Table 2:** Correlation coefficients for days to heading, plant height and percentage of infected heads in the tested 330 genotypes

	Days to heading	Plant height	Infected heads %
Days to heading		−0.15**	−0.11*
Plant height			0.05
Infected heads %			

*p < 0.05, **p < 0.01

### Association mapping for common bunt resistance and some agronomic traits under infection

#### Population structure

Population structure analysis (PS) was performed using 35,216 SNPs after filtering based on a minor allele frequency (MAF > 0.05), missing SNPs < 20% and missing genotypes < 20% [15]. For the association analysis, the heterozygous loci were marked as missing values and the SNP data was re-filtered with the same criteria. As a result, a set of 318 genotypes and 23,053 SNPs were used in our GWAS analysis.

**Fig. 3:**
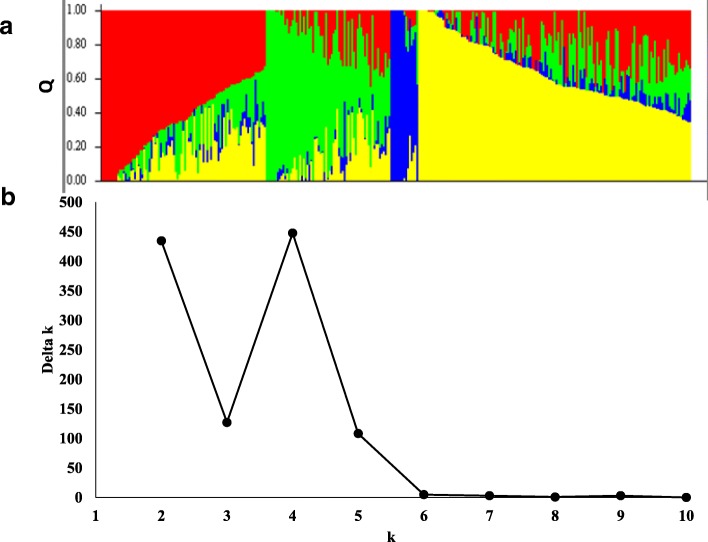
Analysis of population structure using 35,216 SNP markers: (**a**) Estimated population structure of 318 winter wheat genotypes (k = 4). The y-axis is the sub-population membership, and the x-axis is the genotypes. **b** delta-k for different numbers of sub-populations

The PS analysis was carried out on the 318 genotypes (TRP2015 and DUP2014 nurseries) and four possible subpopulations were found (Fig. 3a). To verify this result, the number of proposed k was plotted against the calculated ∆k. A sharp and clear peak was assigned to k = 4 (Fig. 3b). Therefore, four subpopulations was chosen to define the genetic structure of the 318 genotypes.

#### Genome-wide association study (GWAS) for common bunt resistance

**Fig. 4:**
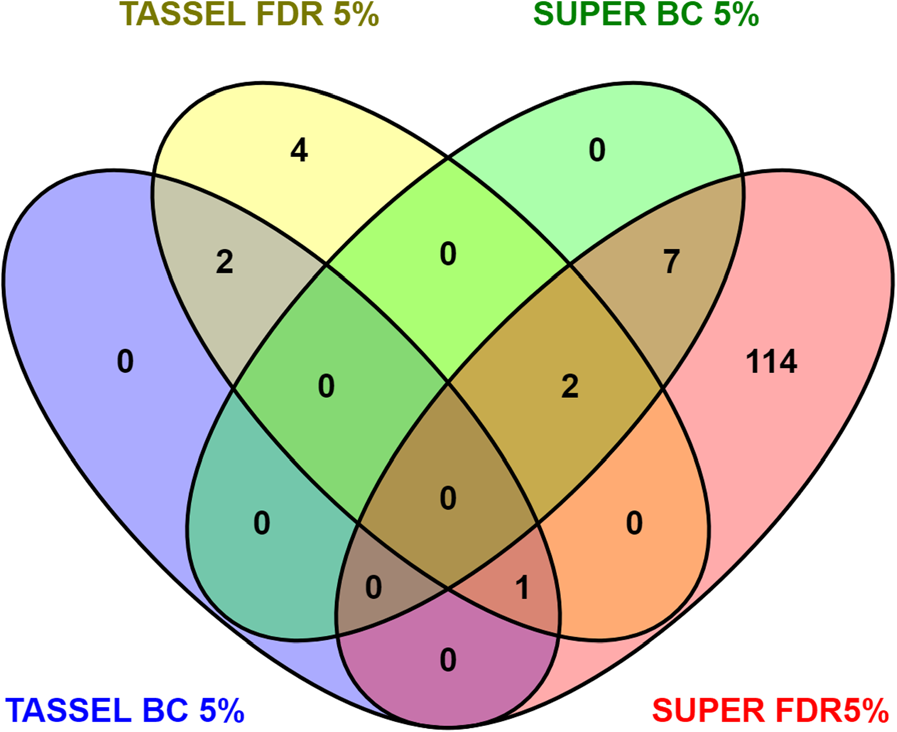
Summary of the significant SNPs associated with common bunt resistance based on TASSEL and SUPER analysis for GWAS detected by Bonferroni correction (BC 5%) and false discovery rate (FDR 5%)

**Fig. 5:**
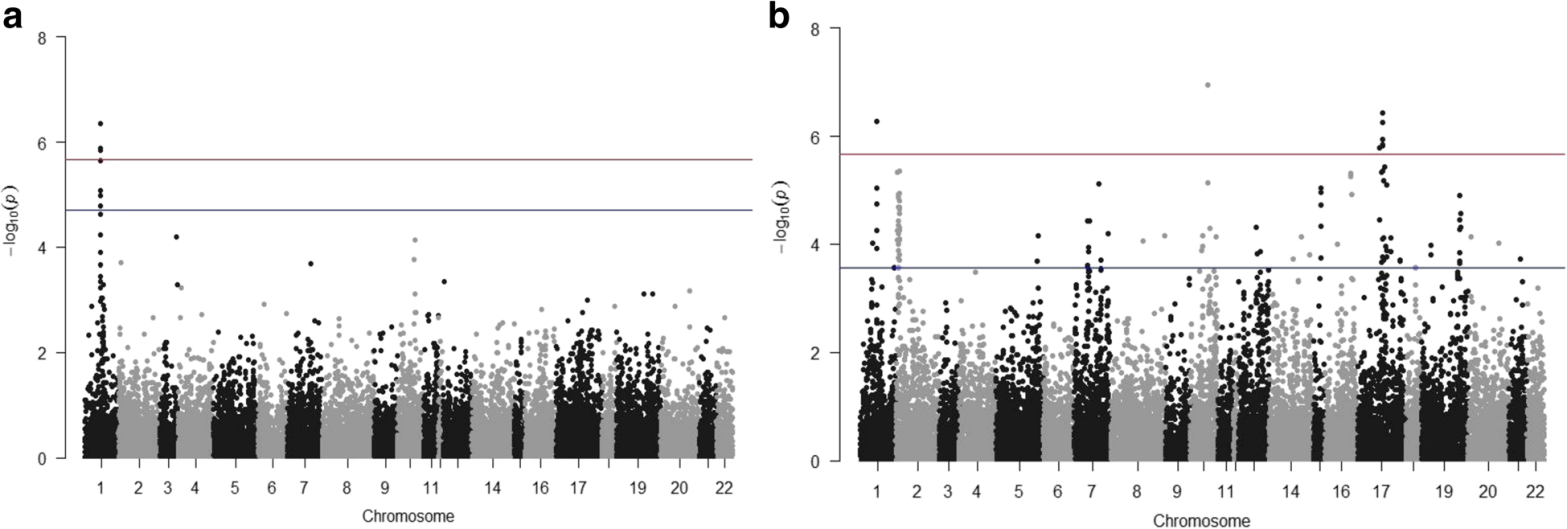
Manhattan plot displaying SNP marker-trait association identified for common bunt resistance in GWAS using 318 winter wheat lines using **a**) TASSEL and **b**) SUPER softwares. Redline is significance threshold of 5% Bonferroni correction while blue line is significant threshold of 5% false discovery rate (FDR). Y axis is the chromosome number where, 1=1A, 2=1B, 3=1D, 4=2A, 5=2B, 6=2D, 7=3A, 8=3B, 9=3D, 10=4A, 11=4B, 12=4D, 13=5A, 14=5B, 15=5D, 16=6A, 17=6B, 18=6D, 19=7A, 20=7B, 21=7D, 22= unknown chromosome

Due to the absence of the LxE interaction for the common bunt resistance, transformed data of the average from Lincoln and Mead were combined and two models of MLM (K) and MLM (Q + K), due to the presence of population structure, in TASSEL, were used. Association analyses, performed by TASSEL 5.0, using both models identified nine SNPs to be associated with common bunt resistance based on FDR (α = 0.05) and only three SNPs based on Bonferroni correction (α = 0.05). All the significant SNPs were located on chromosome 1A (Additional file 4: Table S1). To investigate if there are more genes controlling the common bunt resistance in wheat, the GWAS using SUPER method was done. The SUPER analysis identified nine SNPs, located on three chromosomes, to be associated with common bunt resistance based on a Bonferroni correction (α = 0.05) and a set of 123 significant SNPs located on fourteen chromosomes based on FDR (α = 0.05). A summary of the association results is presented in Fig. 4 and Table 3. Manhattan plot for GWAS results indicated the chromosomal location of the different significant SNPs based on TASSEL and SUPER analysis (Fig. 5a and b).

**Table 3:** Summary of GWAS results using both TASSEL and SUPER methods indicating the presence of the 123 SNPs significantly associated with common bunt resistance in wheat

Chro.	No. of SNPs	Position	*P*-value	R^2^	Allele effect
1A	8	42,100,559–499,864,432	0.0000962–0.0000566	1.0–9.0%	(−0.26) – (− 2.70)
1B	31	137,128,882–163,096,159	0.00000471–0.000026	0.5–5.0%	(− 0.06) – (− 0.29)
2B	2	787,820,195–785,905,982	0.000126–0.00026	1.7 2.9%	(−0.10) – (− 0.12)
3A	10	51,290,068–742,470,499	0.0000366–0.000062	0.6–4.0%	(−0.06) – (− 0.30)
3B	2	6,951,948–851,809	0.0000882–0.000070	0.6–1.0%	(−0.090) – (− 0.097)
4A	8	632,236,000–738,778,127	0.000132015–0.0000744	0.9–5.0%	(−0.07) – (− 0.28)
5A	3	568,046,700–613,546,850	0.000102–0.00021	0.02–2.0%	(−0.02) – (− 0.11)
5B	6	550,732,248–701,156,196	0.0000485–0.000138288	0.7–8.0%	(−0.06) – (− 0.22)
5D	5	544,283,538–545,103,958	0.0000188–0.00018284	2.3–2.9%	(−0.135) – (− 0.143)
6A	5	431,921,921–611,857,844	0.000102731–0.0000121	0.1–5.0%	(−0.01) – (− 0.22)
6B	28	461,408,979–708,256,236	0.0000348–0.000198768	0.1–3.0%	(−0.05) – (− 0.32)
7A	12	298,990,918–722,414,973	0.000155936–0.0000268	1.0–4.0%	(−0.06) – (− 0.13)
7B	2	18,097,824–703,152,031	0.0000718–0.0000972	1.0–4.0%	(−0.17) – (− 0.29)
7D	1	595,913,707	0.000188716	3.1%	−0.17

Based on the summarized results of the GWAS analysis, the phenotypic variation explained by marker (R^2^) for all the significant SNPs ranged from 0.1 to 9.0%. The number of significant SNPs located on the same chromosome ranged from one SNP on chromosome 7D to 31 SNPs on chromosome 1B (Table 3). The detailed GWAS results for each significant marker including *p*-value, R^2^, allele effect, target allele (resistant allele) and LD between each pair of markers located on the same chromosome are presented in Additional file 4: Table S1. Obviously, only five SNPs out of the nine SNPs identified by TASSEL were identified using SUPER. Significant and non-significant LD of different marker pairs were found on the different chromosomes. For example, no LD was found between the significant SNPs on chromosomes 3B, 5A, and 7B, while a complete LD among the 31 significant markers were found on chromosome 1B (Additional file 4: Table S1).

Association analysis for plant height and days to maturity under common bunt conditions was performed. No significant SNPs were found for days to heading using TASSEL, while eight SNPs were found to be associated with plant height under the infection based on a Bonferroni correction and FDR (α = 0.05) (Table 4 and Additional file 5: Figure S4). These significant SNPs located on chromosomes 1A (three SNPs), 4B (two SNPs) and 5B (three SNPs). The phenotypic variations of these significant SNPs ranged from 7.67 to 10.04%.

**Table 4:** Association analysis of plant height under common bunt infection using the mixed linear model (MLM) using TASSEL 5.2 software based on Bonferroni correction (α = 0.05) and false discover rate (α = 0.05)

SNP ID	Chro.	*p*-value	Target allele ^(1)^	Allele effect ^(2)^	R^2 (3)^ (%)
S1A_39183276	1A	8.22E-06	C:T	4.04	7.67
S1A_40498783	1A	1.55E-05	G:T	3.82	7.83
S1A_40498796	1A	1.55E-05	G:A	3.82	7.83
S4B_28403490*	4B	1.45E-06	G:T	5.85	8.75
S4B_36090153	4B	1.44E-05	A:G	5.15	7.88
S5B_533748253*	5B	1.13E-06	A:T	8.36	8.25
S5B_533748257*	5B	1.13E-06	G:C	8.36	8.25
S5B_565504007*	5B	1.19E-06	C:T	8.87	10.04

*SNPs significantly associated with plant height based on Bonferroni correction (α = 0.05)

^(1)^ The allele on the left increased the resistance

^(2)^ The effect of left allele associated with increased resistance

^(3)^ Phenotypic variation explained by marker

### Genes underlying candidate SNPs and their annotations and expression

As there were no promising significant correlations between common bunt resistance and other agronomic traits (plant height and days to heading), we focused on identifying gene candidates for significant SNPs associated with common bunt resistance to further understand this association. The annotation of genes containing these SNPs was reviewed. There were no SNPs located within gene models on chromosomes 1A, 2B, 3A, 4A, 5A, and 7D. However, many SNPs were found within gene models on the remaining chromosomes (Table 5). The functional annotation of these gene models was retrieved using IWGSC v1.0 GFF3 files. Some of these gene models were found to be associated with disease resistance. For example, the seven gene models on chromosome 1B have been found to control disease resistance and increase plant defense against disease and pests (Table 5).

**Table 5:** Gene models underlying significant SNPs and their annotations from the International Wheat Genome Sequencing Consortium reference genome assembly v1.0 of the variety Chinese spring

Chrom.	SNP ID	Gene model	Gene annotation	Probable function	References
1B	S1B_137,128,882	TraesCS1B01G116700.2	Serine/threonine-protein kinase	Plant defense	[49]
		TraesCS1B01G116700.1			
	S1B_144926670	TraesCS1B01G121600.2	1,3-beta glucosidase	Cell division and plant defense	[50]
	S1B_144929898	TraesCS1B01G121600.3			
	S1B_148891914	TraesCS1B01G123200.2	Kinesin-like protein	Plant defense	[51]
		TraesCS1B01G123200.1			
	S1B_160486821	TraesCS1B01G130000.1	Cytochrome P450	Pest and disease resistance	[52]
	S1B_160486833				
3B	S3B_6,951,948	TraesCS3B01G016700.1	RNA polymerase II transcription subunit	Regulation of pathways	[53]
5B	S5B_550,732,248	TraesCS5B01G372800.3	Rhomboid family protein	Regulated protein	
		TraesCS5B01G372800.1			
		TraesCS5B01G372800.2			
5D	S5D_544799149	TraesCS5D01G527700.1	Lytic transglycolase		
6A	S6A_431,921,921	TraesCS6A01G229000.1	SPla/RYanodine receptor (SPRY) domain protein	Increase plant immunity for Nematoda	[54]
	S6A_431921934				
	S6A_611,857,844	TraesCS6A01G406700.1	NAC domain protein	Control biotic and abiotic stresses	[55]
6B	S6B_466695469	TraesCS6B01G258800.1	ORYGL Auxin response factor	Regulate gene expression	[56]
	S6B_507468652	TraesCS6B01G281100.1	Protein of unknown function	–	
	S6B_576206570	TraesCS6B01G326600	Protein of unknown function	–	
		TraesCS6B01G326600.1			
	S6B_590912673	TraesCS6B01G335600.1	WHEAT Hexosyltransferase		
	S6B_590912695				
7A	S7A_714662345	TraesCS7A01G537200	–	–	
		TraesCS7A01G537200.1			
	S7A_715781215	TraesCS7A01G538800.1	–	–	
7B	S7B_18,097,824	TraesCS7B01G020500.1	–	–	

**Fig. 6:**
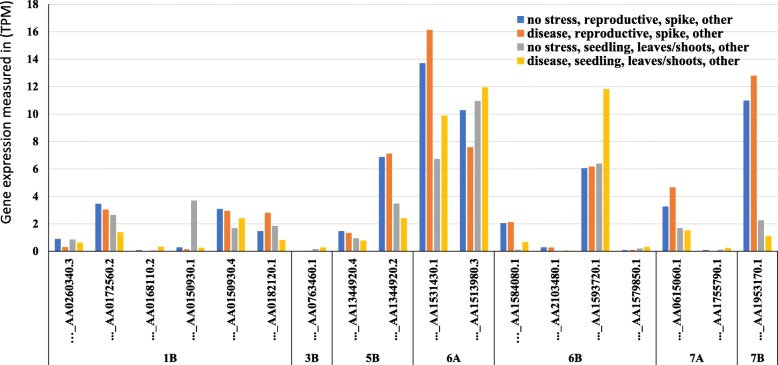
The expression of the gene models harboring significant SNPs in transcripts per million (TPM). Blue and gray columns represent the gene expression under controlled conditions at reproductive and seedling growth stages, respectively. While, orange and yellow columns represent the gene expression under disease infection conditions at the same growth stages

In order to provide more information about the resistance genes in our tested materials, the expression of the identified gene models was investigated and presented in Fig. 6. Comparing among the expression of these genes under control and diseased conditions at seedling and reproductive stages, only nine gene models were found to have higher expression under disease conditions. For example, one gene model was found to have a higher expression at seedling stage and another one at the reproductive stage on chromosome 1B. One gene model were identified to have higher expression under disease conditions on chromosomes 3B, 5B, 7A, and 7B. While both gene models identified on chromosome 6A have a higher expression under disease.

## Discussion

The experiments in both locations were planted on October 14th^,^ 2015. The soil temperature at 10 cm depth was 18 °C and 17 °C in Lincoln and Mead, respectively. Cool soil temperature at the time of planting is favorable for common bunt infection [1]. Beginning in mid of November, snow covered the plots until mid of January. A long period of snow coverage is also important to cause a high level of disease. The evaluation test of common bunt resistance could be considered as a valid test only when the mean percent of the infected spikes in the susceptible checks exceeded 50% [16]. While Heines VII had a lower score, in our experiment, some genotypes had a high degree of susceptibility to common bunt (six genotypes based on the average) with the percent of infected heads exceeded 50% (64.49%, Fig. 1). Based on these numbers of susceptible genotypes, we concluded the recent field test was considered valid. The low value for Heines VII may relate to its being very un-adapted to Nebraska. The high percentage of infected heads in the spring check, Red Bob 73.4%, indicated the highly effective greenhouse inoculation. When the infection percentage of a genotype was less than 10%, the resistance gene which it carries could be considered as an effective gene [17].

### Resistance genes to Nebraska common bunt race

The differential lines were very useful in this study because they shed light on the possible resistant genes which could exist in Nebraska winter wheat genotypes. Based on the results of the differential lines inoculation, we found *Bt6, Bt7, Bt9, Bt10, Bt11, Bt12, Bt13*, *Bt15,* and *Btp* are effective resistance genes for the Nebraska common bunt race which is mainly virulent on *Bt1, Bt2, Bt3, Bt8,* and *Bt14*. The differential lines used in this study were also used in earlier studies to identify the virulence characteristics of wheat bunt isolates [18–21]. These data on differential lines were also very important to target the possible genes that can be used to breed local bunt-resistant cultivars. Two advantages of using these differential lines are (1) they can be highly and morphologically discriminated from each other which leads to more accurate scoring [20] and (2) they are available to the international scientists via the United States Department of Agriculture–Agricultural Research Service, National Small Grains Collection (NSGC) in Aberdeen, ID.

### Genetic variation in common bunt resistance and some agronomic traits

The highly significant differences among the tested genotypes for common bunt resistance indicate that high levels of variation existed within the Nebraska breeding pool. This high genetic variation is very useful for selecting the most resistant genotypes to be used as parents in future Nebraska winter wheat breeding program especially for low input or organic production where seed treatments are not commonly used. In the eastern half of Nebraska, common bunt disease in wheat is found to be frequently occurring but to a varying extent (https://cropwatch.unl.edu/common-bunt-wheat-unl-cropwatch-august-28-2013). Therefore, breeding Nebraska winter wheat for common bunt resistance is needed to avoid yield and quality (due to odors) losses, especially in organic production. The high correlation for common bunt resistance between Mead and Lincoln indicate that the response to common bunt for most of the genotype was similar across the two locations. Moreover, this high correlation is in agreement with non-significant location x entry (LxE) interaction. The high broad-sense heritability value for common bunt resistance indicates that common bunt resistance is a highly heritable trait and selection for a high common bunt resistance will be successful.

The absence of significant differences among the tested genotypes for chlorophyll content under common bunt infection indicates that the infection has a little effect on the chlorophyll content. However, the presence of highly significant differences among the genotypes for plant height and days to flowering under common bunt indicates that genetic variation existed among genotypes under the infection conditions. No significant correlation was found between common bunt resistance and plant height. The correlation between common bunt resistance and agronomic traits was previously tested in two different doubled haploid population [22]. They did not find any correlation between plant height and number of days to heading in one population consisting of 48 lines, while a low significant correlation was found between the resistance and days to heading (0.23*) and plant height (0.24**) in the other population with 115 genotypes. Based on our results and the results of [22] we can conclude that common bunt resistance seems to be an independent trait. The negative correlation between the percentage of infected heads and days to heading indicated that susceptible genotypes are usually heading earlier than resistant genotypes. However, due to the low value of the correlation between days to heading and infected heads, days to heading should not be used as a selection criterion for common bunt resistance in wheat.

### Association mapping for common bunt resistance and some agronomic traits under infection

The number of significant SNPs was greatly increased using SUPER compared with TASSEL. The SUPER has been reported to be a powerful analysis for identifying genes with a smaller effect in any sample size as it extracts a small subset of SNPs and tests them in FaST-LMM. This method increases the statistical power and retains the computational advantages [23]. The low matching of the results of TASSEL and SUPER (only five SNPs) indicating that both methods used together could be beneficial in identifying possible candidate SNPs associated with the studied trait. Summarized results of both GWAS methods identified a set of 123 SNPs significantly associated with the resistance. Due to the low R^2^ of these SNPs (less than 10%), all of them were considered as minor QTLs for common bunt resistance. The different degrees of LD between the significant SNPs on the same chromosome indicates the presence of multiple haplotype blocks on each chromosome, except for chromosome 1B which had a complete LD among its 31 significant SNPs. Hence, chromosome 1B is expected to be carrying a single haplotype block comprising 31 SNPs for common bunt resistance. A set of four SNPs on chromosome 2B and four on chromosome 7A significantly associated with common bunt resistance were identified by [24] using a set of 158 RILs Canadian spring wheat genotyped by 19,639 polymorphic SNPs. They found that the phenotypic variation explained by these markers was 18.7% for the SNPs on chromosome 2B, while it ranged from 10.3 to 20.5% for the SNPs on chromosome 7A. In addition, a set of two QTLs on chromosome 2B and one QTL on chromosome 7A were found to be associated with the resistance in a set of 250 genotypes genotyped by 1824 polymorphic DArT markers in Denmark [25]. These results confirm our results of the presence of resistance genes on chromosomes 2B and 7A. In our study, the number of genotypes (330) and SNPs (23,052) were higher than those used in the previous studies and the SNPs were better distributed across the genome, the resolution of QTL detection was higher than the previous studies (Additional file 6: Figure S5).

**Fig. 7:**
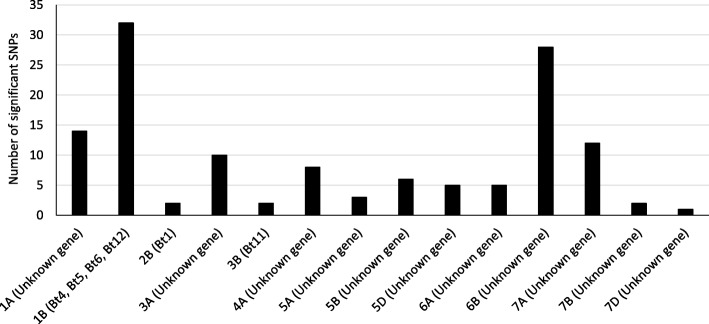
Histogram represents the number of significant SNPs associated with common bunt resistance located on the different chromosomes and the possible genes located on these chromosomes

Little research has been done on the within chromosomal location of the different common bunt resistance genes and little is known on the location of these genes (Table 6 and Fig. 7). Based on the results of association mapping, differentials lines and gene annotations, we expected that the significant SNPs located on chromosome 1B could be associated with some genes such as; *Bt1*, *Bt6*, *Bt12* or other unknown genes [26, 27]. *Bt4* could be located on this chromosome due to the high linkage between it and *Bt6* gene [26]. The significant SNPs on chromosomes 2B and 2D could be associated with *Bt1* and *Bt11* which have been mapped to chromosomes 2B and 2D, respectively [4, 28]. Some QTLs were reported to have an association with common bunt resistance on chromosome 1A [27, 29], but no genes were mapped on this chromosome. The GWAS was performed for other traits (plant height and days to heading). The results indicated the absence of significant QTLs for days to heading under common bunt. However, for plant height, eight SNPs were found to be significantly associated with common bunt infections. The chromosomal locations of these significant SNPs were in agreement with the location of previously reported SNPs and QTLs associated with plant height [30–32]. In addition, some plant height genes were mapped on chromosome 4B such as *Rht1* and *Rht3* [33, 34]. No plant height genes were mapped on the remaining two chromosomes (1A and 5B). By looking at the common markers between plant height and common bunt resistant, the two traits did not have any common markers. This result provides further support for (1) low phenotypic correlations among the traits and (2) common bunt resistant is controlled by an independent genetic system.

**Table 6:** Differential lines used in this study including their PI number, resistance gene, gene location and citation when available

Wheat lines	Resistance Gene	CI or PI number	Chromosomal location of resistance gene	References
Red Bobs	None	CI 6255	–	–
Heines VII	None	PI 209794	–	–
Sel 2092	Bt1	PI 554101	2B	[28]
Sel 1102	Bt2	PI 554097	Unknown	
Ridit	Bt3	CI 6703	Unknown	
Rio	Bt6	CI 10061	1B	[26]
Sel 50,077	Bt7	PI 554100	2D	[14]
PI 173438/ Elgin	Bt8	PI 554120	Unknown	
Elgin/PI 178383	Bt9	PI 554099	6DL	[48]
Elgin/PI 178383	Bt10	PI 554118	6D	[14]
Elgin/PI 166910	Bt11	PI 554119	3B	[4]
PI 119333	Bt12	PI 119333	1B	[27]
Thule III	Bt13	PI 181463	Unknown	
Doubbi	Bt14	CI 13711	Unknown	
Carleton	Bt15	CI 12064	Unknown	
PI 173437	Btp	PI 173437	Unknown

## Conclusion

In conclusion, the high genetic variation among the genotypes is very useful for selection to common bunt resistance in Nebraska wheat. Moreover, differential lines shed the light on the possible genes that may exist in the Nebraska wheat and the virulence of the strain of common bunt found in Nebraska. This result could be useful for crossing the genotypes, as parents, carrying the highest number of resistant genes. The most resistant genotypes identified in this study could be introduced to organic farmers and used for breeding to improve resistance to common bunt in winter wheat. The identified 123 SNPs associated with common bunt resistance in wheat could be a reliable source for marker-assisted selection (MAS) by converting them to Kompetitve Allele-Specific PCR (KASP) markers. However, these SNPs should be validated in a different genetic background before using them for MAS.

## Methods

### Plant material

Three hundred and forty-four wheat genotypes were used in the current study. These genotypes were classified into two sets: differentials lines and tested genotypes.

The differential lines consisted of 14 common bunt differential lines which were used to identify the virulence of the Nebraska common bunt race (Table 6). These lines are used world widely and were obtained from the USDA-ARS. The differential lines contained genes *Bt1* through *Bt13* and *Btp* are winter hexaploid wheat, while, the differential lines for *Bt14* and *Bt15* genes are spring tetraploid (*T. durum* L.) wheat. In addition, two susceptible lines Heines VII “winter wheat” and Red Bobs “spring wheat” were included in this experiment to determine the disease pressure in the field and the greenhouse. The two susceptible lines are part from the worldwide differential lines. The tested genotypes consisted of two populations; 270 winter wheat genotypes from the 2015 F_3:6_ nurseries (Nebraska Duplicate Nursery- DUP2015, the preliminary yield trial). These genotypes were derived from 800 to 1000 crosses. In addition, 60 genotypes from the 2015 F_3:7_ nurseries (Nebraska Triplicate Nursery-TRP2015, the advanced yield trial) which is derived from the selections from the DUP2014 nursery based on the grain yield, grain weight, resistance to disease, end-use quality, plant height and maturity traits and do not overlap with the DUP2015 [35]. The DUP2015 and TRP2015 were developed by the University of Nebraska where P.S. Baenziger is the responsible wheat breeder. This germplasm is officially owned by the Board of Regents, University of Nebraska and is freely available for research purposes within the University of Nebraska by its faculty, students, and visiting scientists.

### Common bunt inoculation

The seeds of all genotypes were inoculated using the method of [16] by mixing the kernels with the teliospores, putting them in an envelope and shaking until the kernels were fully covered with the spores. This method was reported as an effective method to inoculate small amount of seeds, from five to twenty grams.

### Experimental layout

The spring differential lines and spring susceptible check were planted in the greenhouse in five replications under controlled conditions using randomized complete block design. The twelve winter differential genotypes were evaluated in the greenhouse (along with the spring differential lines) in three replications and in the field experiments (along with winter wheat genotypes) in two replications to make sure that none of the genotypes escaped from infection under field conditions. The greenhouse experiment included the spring susceptible check “Red Bob” in order to confirm the success of the inoculation method. All the tested genotypes were placed in the vernalizer for two months at 4 °C with 12 h. of low light to provide optimal conditions for the fungal spores to infect the seedlings and have a high level of infection. The inoculated plants were transferred to a warmer room at 16 °C [night] - 25 °C[day] and grown using an increasing long day (from 12 to 16 h of supplemental Light) to maturity at which time when they were harvested and scored [16].

The two tested nurseries (DUP2015 and TRP2015, had a total of 330 genotypes) were tested in the field. The experiments were conducted in season 2015/2016 at two locations Mead and Lincoln, Nebraska, USA. The experimental design was replicated augmented incomplete block design with three replications and five incomplete blocks each. Two checks ‘Goodstreak’ and ‘Freeman’ were included three times in each block (15 times in each replication). The inoculated seeds of each genotype were sown in a one-meter long row at depth 5 cm. Each row was planted in a group of four with 30 cm between rows. The planting date was October 14th, 2015 and the soil temperature was 18 °C and 17 °C at 10 cm depth at Lincoln and Mead, respectively (http://hprcc.unl.edu/). In each experiment, a winter susceptible line (Heines VII) were included to verify the effectiveness of the inoculation.

The following traits were recorded on each genotype at Lincoln and Mead: days to heading (measured by calculating the number of days after Jan. 1 to when 50% of the tillers in each genotype were at Feekes stage 10.1 and had heads fully emerged from the boot), average chlorophyll content from five flag leaves (Feekes stage 10.5, measuring using SPAD-502 m (KONICA MINOLTA, New York, USA; [36]), and plant height (measured during ripening stage (Feekes stage 11) as the height of the plant from the ground to the tip of the head, awns excluded). In addition, common bunt resistance was measured on each genotype in each replication as follows:

$$ CB=\frac{number\ of\ infected\ heads}{total\ number\ of\ heads/ genotype}\times 100 $$

The level of resistance was determined using the following scale: Percentage of infected heads 0.0% = very resistant, 0.1–5.0% = resistant, 5.1–10.0% = moderately resistant, 10.1–30.0% = moderately susceptible, 30.1–50.0% = susceptible, 50.1–100.0% = very susceptible [37]**.** Data of the different traits were collected using field book Android application [38]. In each location, all traits were scored in three replications except common bunt resistant which was scored in two replications due to a labor involved with counting and scoring all the tillers of a number of genotypes.

### Statistical analysis of common bunt resistance and the studied traits

To improve normality of the common bunt resistance data, the data were transformed using arcsine root square method using Excel 2013 as it was estimated as a percentage. Shapiro-Wilk normality test was used to confirm the improved normality of the transformed data compared with the original data. For all the other studied traits, data from Lincoln and Mead experiments were combined and analyzed using SAS Version 9 [39]. The analysis of variance (ANOVA) model was:

$$ \mathrm{Y}=\mathrm{L}+\mathrm{R}\left(\mathrm{L}\right)+\mathrm{Iblock}\ \left(\mathrm{R}\right)+\mathrm{Pcol}\left(\mathrm{Iblock}\right)+\mathrm{Prow}+\mathrm{E}+\mathrm{L}\ \mathrm{x}\ \mathrm{E}+\mathrm{E}\mathrm{rror} $$

Where Y is the observation of genotype, L is location, R(L) is replication nested within locations, Iblock (R) is Iblock nested within replication, Pcol (Iblock) is the number of columns nested within Iblock, Prow is the row number, E is Entry and LxE is location x Entry interaction.

The graphical presentation of box plots for all studied traits was created using R package ‘ggplot2’ [40] and the histograms were created using Excel 2013. Correlation between different traits was calculated using SAS JMP software [41]. The broad sense heritability (H^2^) was calculated across locations using the following formula:

$$ {H}^2={\sigma}_G^2/\left({\sigma}_G^2+\frac{\sigma_{LxE}^2}{L}+\frac{\sigma_e^2}{LR}\right) $$

where ${\sigma}_G^2,{\sigma}_{LxE}^2$ and ${\sigma}_e^2$ are the variance of the lines and the residuals, R is the number of replicates within the location and L is the number of locations.

### DNA extraction and genotyping-by-sequencing (GBS)

DNA was extracted from all the 330 tested genotypes (270 and 60 genotypes in the DUP2015 and TRP2015, respectively) using BioSprint 96 DNA Plant Kits (Qiagen, Hombrechtikon, Switzerland) from 2 to 3 leaves of two-week-old seedlings. Two restriction enzymes, *PstI* and *MspI* were used to digest the extracted DNA [7]. The sequencing of the pooled libraries was done using Illumina, Inc. NGS platforms. SNP identification was done using TASSEL 5.0 v2 [42]. The reference genome was Chinese Spring genome from the International Wheat Genome Sequencing Consortium (IWGSC) Reference Sequence v1.0 as it was extensively described in [43]. The generated SNP markers were filtered using the following criteria, minor allele frequency (MAF > 0.05), maximum missing sites per SNP < 20% and maximum missing sites per genotype < 20% [15]. The heterozygous loci were marked as missing to avoid overestimation of allele effects (Peter Bradbury, personal communication). Then, the SNP markers were filtered again using the aforementioned criteria. The differential lines were not genotyped using GBS method. Therefore, the differential lines were not used for population structure or genome-wide association analyses.

### Population structure

SNP markers data from both nurseries with a total number of 318 unique genotypes (without differential lines) were used to analyze population structure using the Bayesian model-based software program STRUCTURE 3.4 [44]. The burn-in iteration and Markov chain Monte Carlo (MCMC) replications were set to 100,000. The admixture and allele frequencies correlated models were including in structure analysis. The number of impended iterations was five. The hypothetical number of subpopulations (k) ranged from 1 to 10. The best k was determined using STRUCTURE HARVESTER [45].

### Genome-wide association study (GWAS) and linkage disequilibrium (LD)

Genome-wide association analysis was performed to test marker-trait association for all studied traits in the 318 genotypes (without differential lines). The transformed data of the common bunt resistance was used in GWAS. The GWAS between the SNP markers and all traits was carried out using TASSEL 5.0 software [42]. Two methods were used in GWAS analysis, mixed linear model (MLM (K)) and mixed linear model + Q-matrix (MLM (K + Q)): [46]). The marker-trait association was tested against Bonferroni corrections and false discovery rate (FDR) at a significance level of 5%. The effects of allele of each marker were calculated to determine the influence of the allele on the phenotype. For the common bunt resistance trait, negative allele indicates resistance, while, positive allele indicates susceptible to common bunt. For all other traits, positive allele effects increase the trait values and negative values decrease the trait values. The phenotypic variation explained by a marker (R^2^) was calculated using TASSEL 5.0 [42]. The results of GWAS were presented and visualized suing Q-Q and Manhattan plots using ‘qqman’ R package [47]. For the SNPs located on the same chromosome, linkage disequilibrium (r^2^) among the significant SNPs was calculated by TASSEL 5.0 and illustrated using Excel 2013.

For common bunt resistance, an additional genome-wide association study was done using Settlement of MLM Under Progressively Exclusive Relationship (SUPER) method by GAPIT-R package [23]. SUPER method conducted GWAS by extracting a small subset of SNPs and test them for their association with the target trait by using Fast-LMM. Based on this technique, the SUPER method enabled us to identify minor genes controlling common bunt resistance in the tested nursery.

### Candidate genes and gene annotation

Significant SNPs were inspected as to whether they are in genes identified and annotated in the reference genome assembly (IWGSC Ref Seq v1.0) to further explain the GWAS results. Functional annotation of the genes having significant SNPs was retrieved from the genome annotations provided by IWGSC and examined for their association with disease resistance. For additional understanding of the GWAS results, the gene expression in the different developing stages of wheat was compared based on the wheat expression database (http://www.wheat-expression.com/).

## Additional files

Additional file 1: **Figure S1**. Comparing between the distribution of common bunt resistance scores for the original data (a) and the transformed data using arcsine root square (b). (PDF 217 kb)

Additional file 2: **Figure S2**. Scatter plot represents the correlation between the percentage of common bunt infected heads in the tested genotypes at the two locations (Mead and Lincoln). (PDF 30 kb)

Additional file 3: **Figure S3**. The distribution of a) days to heading and b) plant height under common bunt infection. In the upper part of the figure, histogram represents the frequency of the studied traits as an average of both locations (Mead and Lincoln). In the lower part box plot comparing between the values of the studied traits in the resistance and susceptible genotypes as an average of both locations. (PDF 90 kb)

Additional file 4: **Table S1.** Association analysis of common bunt resistance based on TASSEL and SUPER identified 123 significant SNPs. **Table S2**. List of gene models underlying significant SNPs based on IWGSC and TAGC databases. (XLSX 39 kb)

Additional file 5: **Figure S4**. a) Manhattan plot displaying SNP marker-trait association identified for plant height in GWAS using 318 winter wheat lines. Redline is significance threshold of 5% Bonferroni correction and blue line is significance threshold of 5% FDR. Chromosomes with names written in red are carrying SNPs significantly associated with plant height. b) Quantile-Quantile (QQ) plot used to evaluate the performance of the mixed linear model used for of GWAS for plant height using mixed linear model (MLM + Q-matrix). (PDF 99 kb)

Additional file 6: **Figure S5.** The distribution of the SNPs across a) the different wheat chromosomes and b) the different wheat genomes. Black charts representing the number of SNPs used in the present study while red charts represent the number of SNPs used in previous studies (Lara 2017). (PDF 14 kb)

## Abbreviations

GBS: Genotyping by sequencing
GWAS: Genome-wide association study
KASP: Kompetitve Allele-Specific PCR
LD: Linkage disequilibrium
MAS: Marker-assisted selection
MLM (Q + k): Mixed linear model with Q-matrix + kinship
MLM: Mixed linear model
SNP: Single nucleotide polymorphism
SUPER: Settlement of MLM Under Progressively Exclusive Relationship
