# Supplementary material for: Genetic architecture of common bunt resistance in winter wheat using genome-wide association study
Source: BMC Plant Biol. 2018 Nov 13;18:280. doi: 10.1186/s12870-018-1435-x (PMC6234641; doi:10.1186/s12870-018-1435-x)
Supplement: Supplementary file 1 — Figure S1. Comparing between the distribution of common bunt resistance scores for the original data (a) and the transformed data using arcsine root square (b). (PDF 217 kb) [file 12870_2018_1435_MOESM1_ESM.pdf]

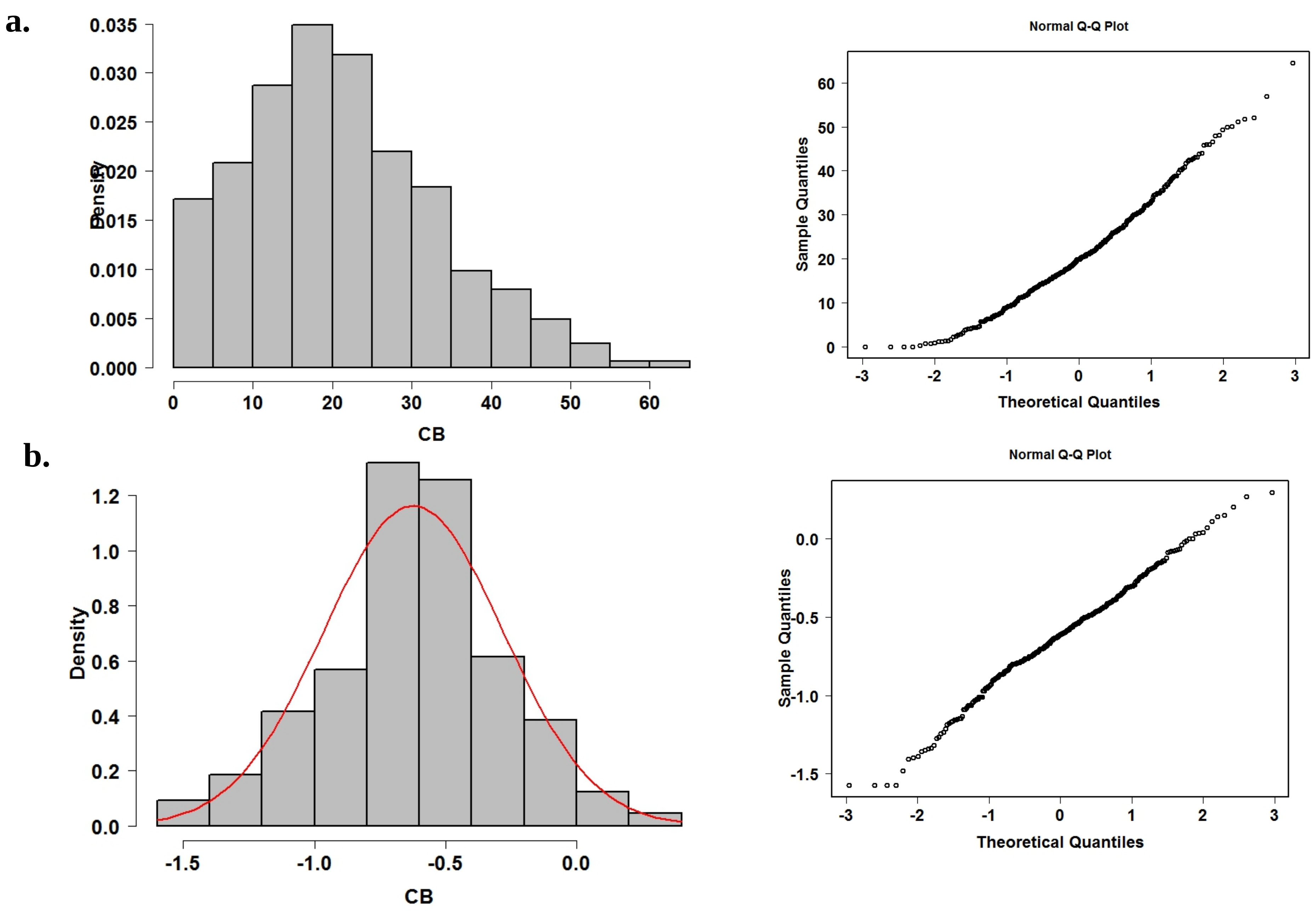

Supplementary figure 1. Comparing between the distribution of common bunt resistance scores for the original data (a) and the transformed data using arcsine root square (b).
