# Supplementary material for: Genetic architecture of common bunt resistance in winter wheat using genome-wide association study
Source: BMC Plant Biol. 2018 Nov 13;18:280. doi: 10.1186/s12870-018-1435-x (PMC6234641; doi:10.1186/s12870-018-1435-x)
Supplement: Supplementary file 2 — Figure S2. Scatter plot represents the correlation between the percentage of common bunt infected heads in the tested genotypes at the two locations (Mead and Lincoln). (PDF 30 kb) [file 12870_2018_1435_MOESM2_ESM.pdf]

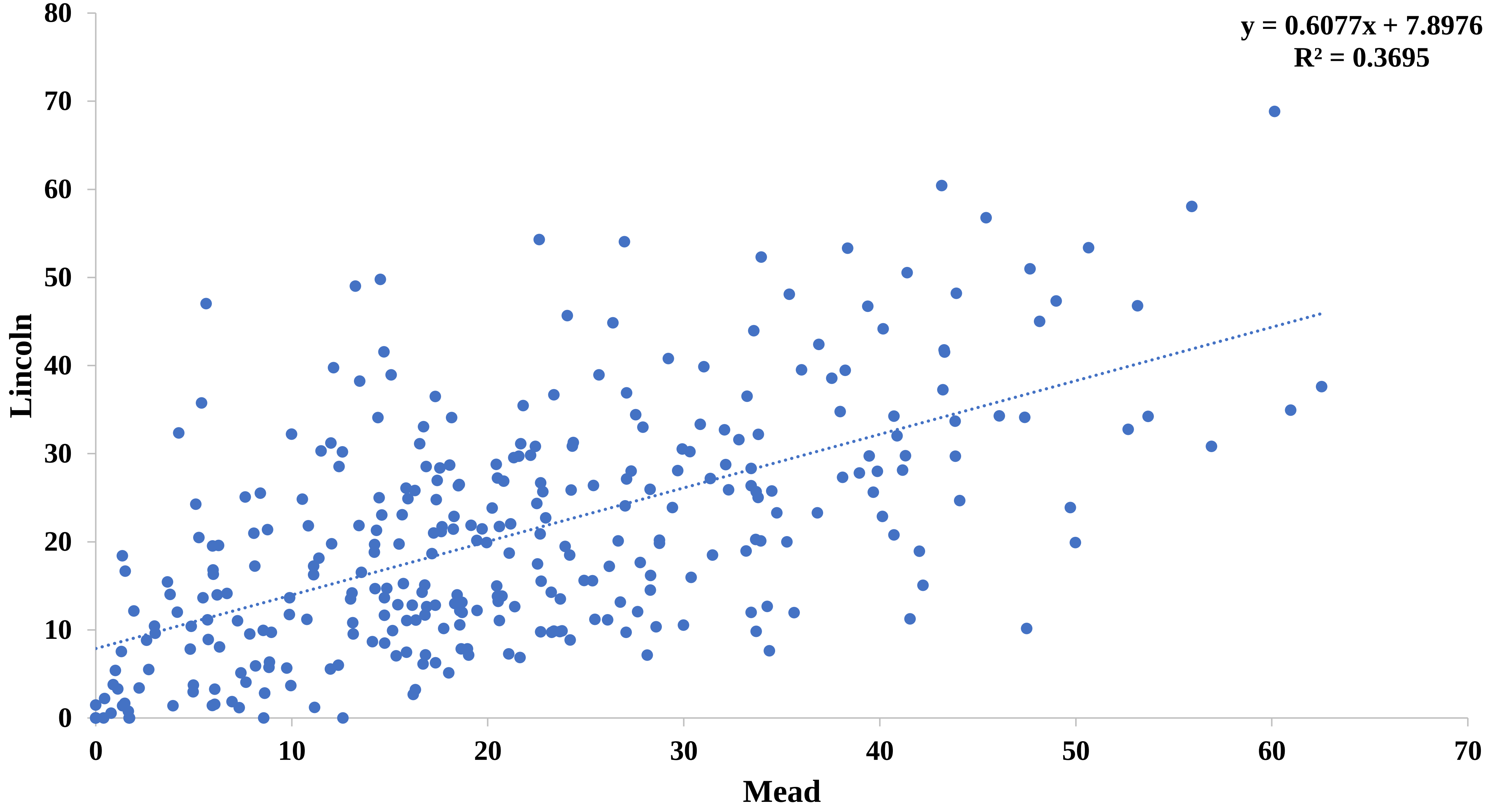

Supplementary figure 2. Scatter plot represents the correlation between the percentage of common bunt infected heads in the tested genotypes at the two location (Mead and Lincoln).
