# Supplementary material for: Genetic architecture of common bunt resistance in winter wheat using genome-wide association study
Source: BMC Plant Biol. 2018 Nov 13;18:280. doi: 10.1186/s12870-018-1435-x (PMC6234641; doi:10.1186/s12870-018-1435-x)
Supplement: Supplementary file 3 — Figure S3. The distribution of a) days to heading and b) plant height under common bunt infection. In the upper part of the figure, histogram represents the frequency of the studied traits as an average of both locations (Mead and Lincoln). In the lower part box plot comparing between the values of the studied traits in the resistance and susceptible genotypes as an average of both locations. (PDF 90 kb) [file 12870_2018_1435_MOESM3_ESM.pdf]

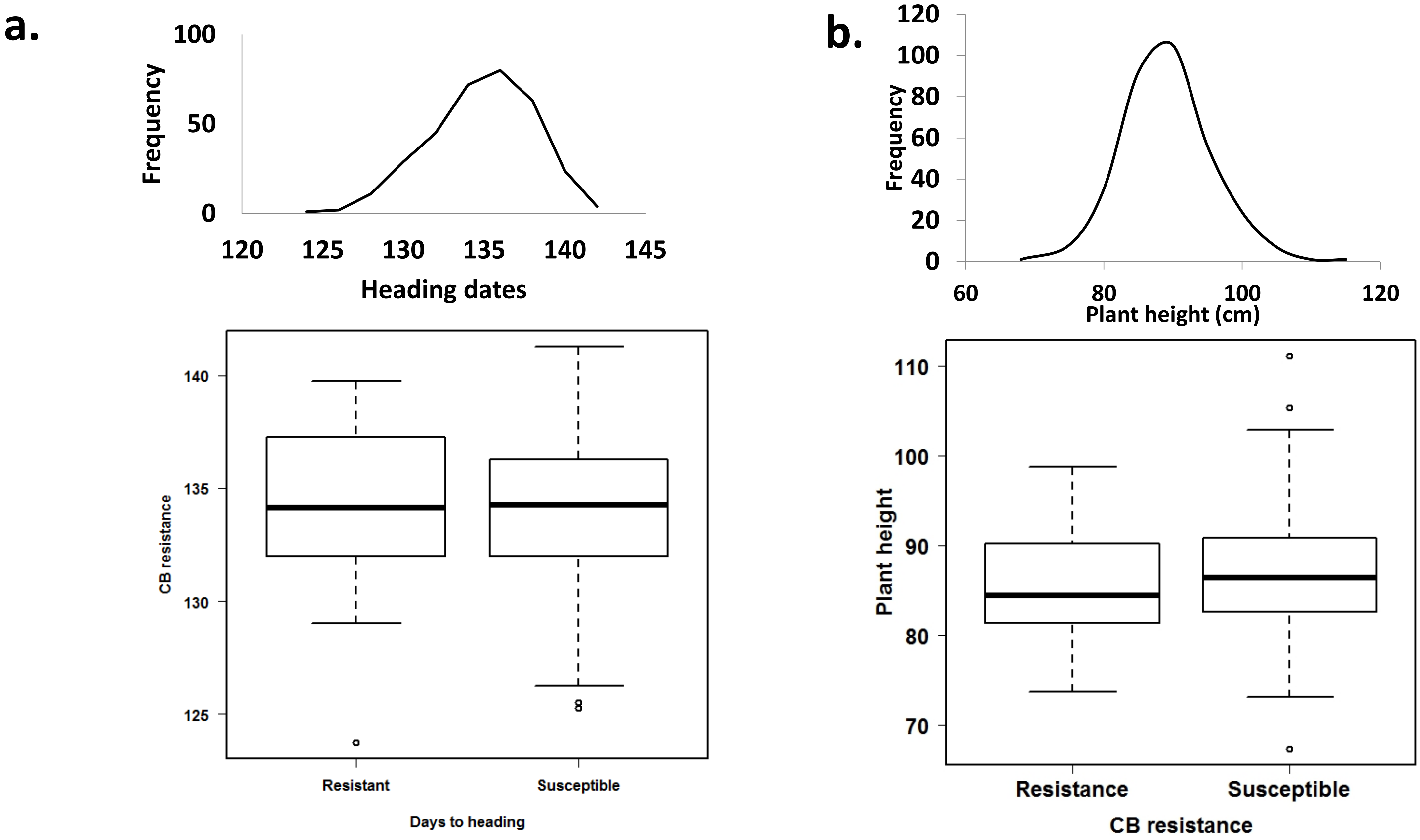

Supplementary Figure 3. The distribution of a) days to heading and b) plant height under common bunt infection. In the upper part of the figure, histogram represents the frequency of the studied traits as an average of both locations (Mead and Lincoln). In the lower part box plot comparing between the values of the studied traits in the resistance and susceptible genotypes as an average of both locations
