# Supplementary material for: Genetic architecture of common bunt resistance in winter wheat using genome-wide association study
Source: BMC Plant Biol. 2018 Nov 13;18:280. doi: 10.1186/s12870-018-1435-x (PMC6234641; doi:10.1186/s12870-018-1435-x)
Supplement: Supplementary file 5 — Figure S4. a) Manhattan plot displaying SNP marker-trait association identified for plant height in GWAS using 318 winter wheat lines. Redline is significance threshold of 5% Bonferroni correction and blue line is significance threshold of 5% FDR. Chromosomes with names written in red are carrying SNPs significantly associated with plant height. b) Quantile-Quantile (QQ) plot used to evaluate the performance of the mixed linear model used for of GWAS for plant height using mixed linear model (MLM + Q-matrix). (PDF 99 kb) [file 12870_2018_1435_MOESM5_ESM.pdf]

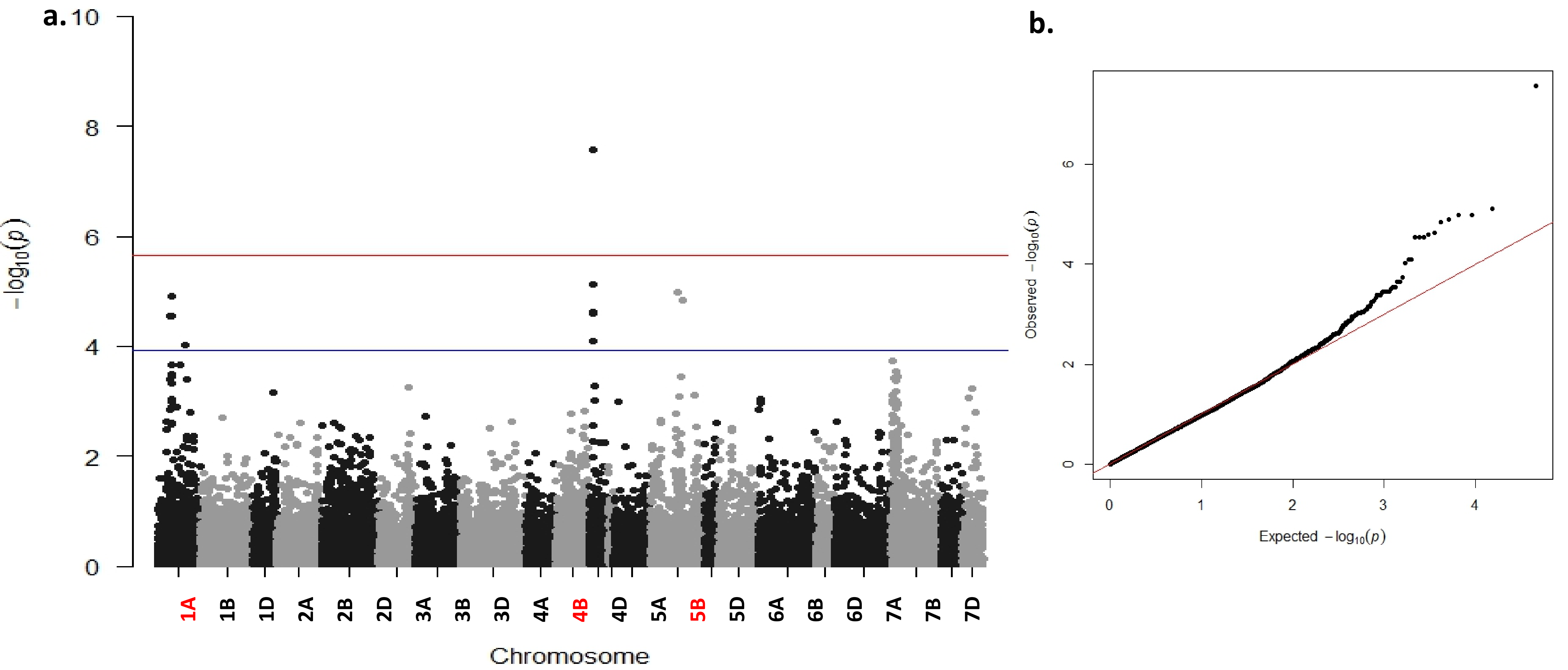

Supplementary Figure 4. a) Manhattan plot displaying SNP marker-trait association identified for plant height in GWAS usin
winter wheat lines. Redline is significance threshold of 5% Bonferroni correction and blue line is significance threshold c
FDR . Chromosomes with names written in red are carrying SNPs significantly associated with plant height. b) Quantile-Qu
(QQ) plot used to evaluate the performance of the mixed linear model used for of GWAS for plant height using mixed linear r
(MLM+Q-matrix).
