# Supplementary material for: Genetic architecture of common bunt resistance in winter wheat using genome-wide association study
Source: BMC Plant Biol. 2018 Nov 13;18:280. doi: 10.1186/s12870-018-1435-x (PMC6234641; doi:10.1186/s12870-018-1435-x)
Supplement: Supplementary file 6 — Figure S5. The distribution of the SNPs across a) the different wheat chromosomes and b) the different wheat genomes. Black charts representing the number of SNPs used in the present study while red charts represent the number of SNPs used in previous studies (Lara 2017). (PDF 14 kb) [file 12870_2018_1435_MOESM6_ESM.pdf]

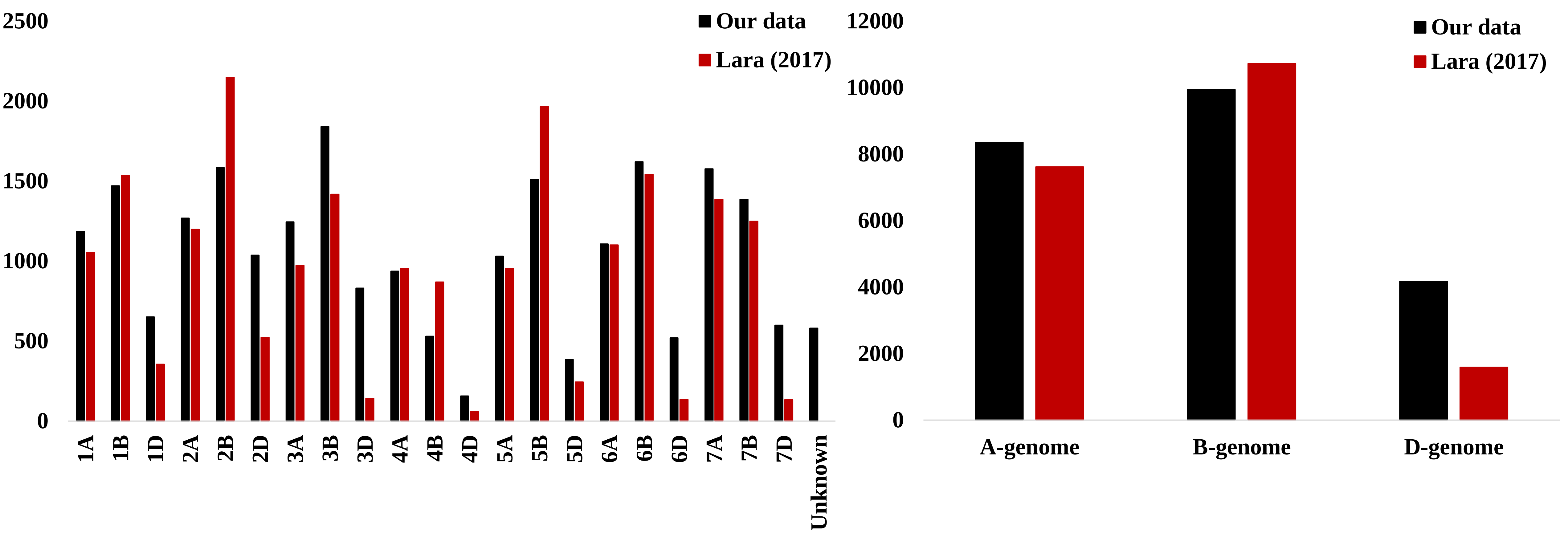

Supplementary Figure 5. The distribution of the SNPs across a) the different wheat chromosomes and b) the different wheat genomes. Black charts representing the number of SNPs used in the present study while red charts represent the number of SNPs used in previous studies (Lara 2017).
